# Assessing the causal relationship between nitrogen dioxide air pollution and schizophrenia risk: A large-scale Mendelian randomization study

**DOI:** 10.1097/MD.0000000000049102

**Published:** 2026-06-05

**Authors:** Chunying Li, Xiao Zou

**Affiliations:** a College of Resources and Environmental Engineering, Guizhou University, Guiyang 550025, Guizhou, China; b Department of Ecology, College of Life Sciences, Guizhou University, Guiyang, Guizhou, China.

**Keywords:** genome-wide association studies, Mendelian randomization, nitrogen dioxide, schizophrenia

## Abstract

Nitrogen dioxide (NO_2_) is mainly discharged from the burning of fossil fuels and remains suspended in the air with other particulate pollutants, which has a significant impact on the Earth’s ecological environment and is harmful to human health. Schizophrenia is a nervous system disease involving emotion, thinking, and behavior. There is no consistent conclusion about the etiology of schizophrenia, though numerous studies are ongoing. Previous studies suggest that exposure to NO_2_ air pollution may increase the risk of schizophrenia, though this remains in the early exploratory stages. We conducted a 2-sample Mendelian Randomization study to investigate the potential causal effect of NO_2_ exposure on schizophrenia risk. Genetic instruments were selected from large-scale genome-wide association studies of European ancestry, including NO_2_ exposure (n = 456,380) and schizophrenia (n = 640,808). To ensure the reliability of our findings, we also conducted sensitivity analyses. Across all Mendelian Randomization models, NO_2_ exposure showed a significant positive causal effect on schizophrenia risk. Beta values ranged from 0.246 (weighted median estimator model, 102 single nucleotide polymorphisms) to 0.478 (inverse variance weighting model, 128 single nucleotide polymorphisms), with all *P* < .05. odds ratios ranged from 1.30 (95% confidence interval: 1.03–1.65, weighted median estimator, exposure: ukb-b-9942; outcome: ieu-b-5099) to 1.60 (95% confidence interval: 1.39–1.87, inverse variance weighting fixed effects, exposure: ukb-b-5620; outcome: ieu-b-5099). Sensitivity analyses and heterogeneity tests confirmed the robustness of these findings. These findings help further our understanding of the etiology of schizophrenia and provide a new perspective for air pollution mitigation.

## 1. Introduction

Nitrogen dioxide (NO_2_) is a reddish-brown irritating atmospheric pollutant discharged from the microbial decomposition of nitrates in nature, biomass combustion, atmospheric lightning oxidation, fossil fuel combustion, and vehicle exhaust emissions.^[1]^ As a precursor substance of ozone and photochemical smog, NO_2_ usually exists in the air along with particulate matter_2.5_ and particulate matter_10_ particle pollutants, which significantly impact the earth’s ecological environment and human health.^[2]^ There is strong evidence that NO_2_ stimulates the respiratory tract, leading to respiratory problems such as asthma and cough.^[2]^ Moreover, long-term exposure to NO_2_ may increase the risk of chronic respiratory and cardiovascular diseases. Additionally, NO_2_ can also increase susceptibility to infections by interfering with the body’s immune system. Notably, recent work has suggested a potential link between NO_2_ and neurological disorders.^[3]^

Schizophrenia is a neurological disease with complex symptoms, characterized by obvious disorders in many mental activities such as cognition, emotion, thinking, and volitional behavior.^[4,5]^ The disease generally diminishes the patient’s social function, and is usually accompanied by serious concurrent diseases.^[6]^ Owing to mental disorder, patients often have a high risk of disability, accompanied by a lifetime suicide risk of 10%, which brings a huge burden to the family and social harmony.^[7]^ The pathogenesis of schizophrenia remains unclear, and some studies suggest that it results from multiple factors, including genetic, psychosocial, neurodevelopmental, and environmental factors, among which environmental factors are a research hotspot.^[8–11]^

With the increase in air pollution worldwide, the problem of ambient air pollution has been increasingly studied. A systematic review and meta-analysis including 12 studies and over 1.2 million participants reported that NO_2_ exposure increases schizophrenia risk.^[12,13]^ A Danish study reported that living in an NO_2_ air pollution environment during childhood may increase the risk of schizophrenia.^[14]^ Another meta-analysis of existing studies confirmed the association between NO_2_ exposure and schizophrenia risk.^[15]^ Furthermore, another study also found a correlation between NO_2_ air pollution exposure and schizophrenia.^[16]^

However, the causal relationship between NO_2_ air pollution exposure and schizophrenia is still in the preliminary stage of exploration. To address this gap, we applied a 2-sample Mendelian randomization (MR) framework using publicly available genome-wide association study (GWAS) summary statistics. MR leverages genetic variants as instrumental variables to estimate causal effects, reducing confounding and reverse causation.^[17,18]^ Here, NO_2_ air pollution exposure is considered the risk factor, and single nucleotide polymorphisms (SNPs) are used as instrumental variables to assess its potential causal effect on schizophrenia. We selected GWAS data on schizophrenia and air pollutants from global authoritative databases. After quality control, we chose genetically independent SNPs associated with NO_2_ exposure and matched them with the schizophrenia GWAS results. To ensure the reliability of our findings, we performed MR analysis using 3 calibration methods and validation checks.

## 2. Methods

### 2.1. Study design

**Figure 1.**
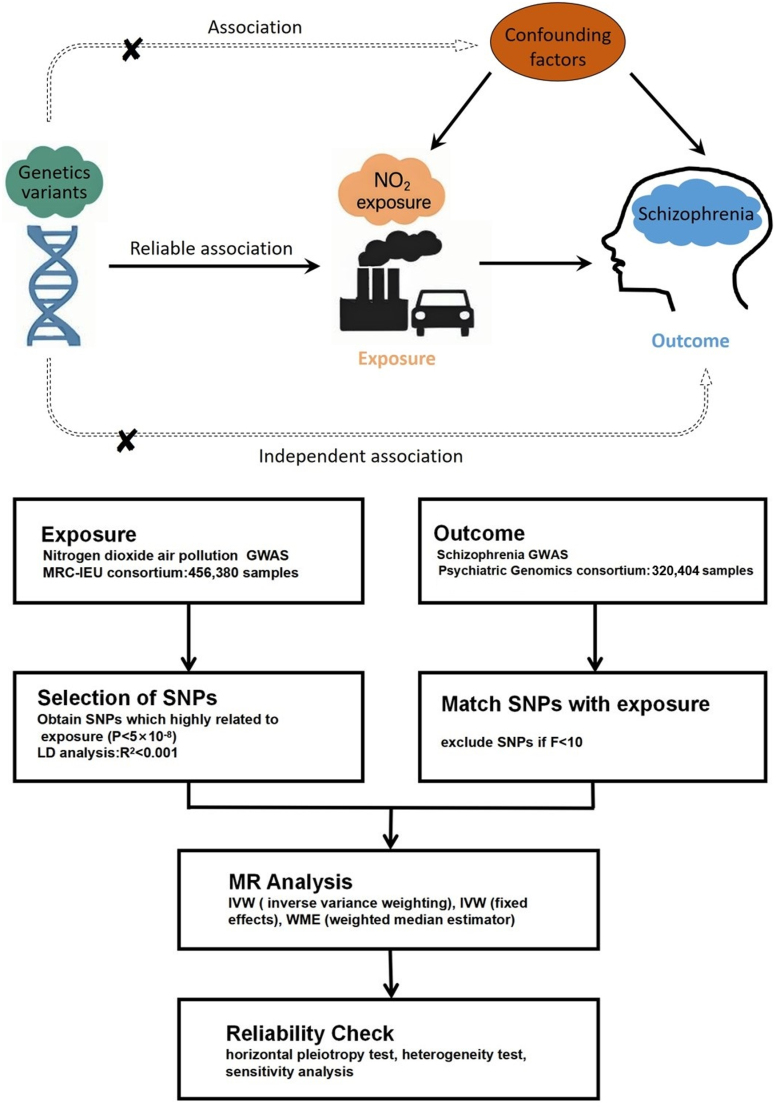
Overview of the MR analysis procedure of the causal effect of NO_2_ air pollution exposure levels on schizophrenia risk. SNPs associated with NO_2_ exposure were first selected based on genome-wide significance (*P* < 5 × 10^−^8) and *F*-statistic > 10 to ensure instrument strength. Independent SNPs were retained using LD clumping (*R*^2^ < 0.001 within 10,000 kb). Selected SNPs were harmonized with schizophrenia GWAS data. MR analysis was performed using IVW, IVW fixed effects, and WME models. Sensitivity analyses, including horizontal pleiotropy assessment, leave-one-out tests, and heterogeneity evaluation, were conducted to confirm robustness. *F*-statistic is the measure of instrument strength. GWAS = genome-wide association study, IVW = inverse variance weighting, LD = linkage disequilibrium, MR = Mendelian randomization, NO_2_ = nitrogen dioxide, SNPs = single nucleotide polymorphisms, WME = weighted median estimator.

This study performed a 2-sample univariate MR analysis. We utilized NO_2_ air pollution levels as an exposure factor and schizophrenia as the outcome measure. In the correlation setting, items with the threshold value *P* < 5 × 10^−8^ were selected. Then, with the clumping function linkage disequilibrium (LD) (*R*^2^ < 0.001) of independent setting and the threshold value *F* > 10 of statistical strength setting, we excluded the useless SNPs. Through a series of quality control measures, the instrumental variables of MR were selected. After selecting the instrumental variables, we conducted causal effect analysis using a 2-sample MR approach, applying inverse variance weighting (IVW), IVW with fixed effects, and the weighted median estimator (WME) models. We also performed reliability analysis (sensitivity analysis, horizontal pleiotropy, and heterogeneity evaluations) to ensure the robustness of the results (Fig. 1).

### 2.2. Data sources

In this study, common atmospheric pollutants were considered as exposure risk factors. To select the relevant genetic variations of these corresponding exposure risk factors, we searched the database of IEU OPEN-GWAS with the keywords: “Sulfur-dioxide,” “Ozone,” “Methane,” “Benzene,” “Nitrogen-dioxide,” “Polychlorinated-biphenyl,” “Chlorofluorocarbon,” “Ammonium,” “Methyl Aldehyde,” “Dioxin,” “Chlorine,” and “Polyaromatic-hydrocarbon.” The IEU OPEN-GWAS which published genome-wide association analyses of human traits, is the largest and most complete public resource.^[19]^ Finally, we identified 139, 128, and 102 NO_2_ air pollution exposure level-related SNPs in 3 different experimental exposure groups from a 456,380-sample size European ancestry GWAS. The GWAS data for the NO_2_ air pollution exposure risk factor was obtained from the Medical Research Council (MRC) Integrative Epidemiology Unit at the University of Bristol, which has been organized into 3 versions by the OpenGWAS database. For this study, we selected the 2 largest sample versions. The schizophrenia GWAS data was sourced from the Psychiatric Genomics Consortium, with core funding from the National Institute of Mental Health (United States of America).

### 2.3. Selecting and filtering the instrumental variables

Based on the threshold value of significant correlation *P* < 10^−5^, we selected 3 sets of instrumental variables (139, 128, and 102 NO_2_ air pollution exposure level-related SNPs) and further excluded the non-biallelic SNPs. Then, we matched the residual SNPs to the schizophrenia GWAS results, during which we tried to align strands of the palindromic SNPs to achieve allele harmonization. In order to ensure mutual independence between the instrumental variables, we conducted an LD analysis, following which the nonindependent SNPs were filtered based on the significance threshold, that is, *R*^2^ < 0.001 within the 10,000 kb window. If there was no NO_2_ air pollution exposure level-related SNP in the schizophrenia GWAS results, we planned to use the proxy SNPs to replace it through LD tagging (*R*^2^ = 1). The filtered SNPs were integrated with the GWAS results of NO_2_ air pollution exposure levels and schizophrenia as instrumental variables.

### 2.4. Statistical analysis

In this study, we conducted a 2-sample univariate MR analysis using the web server “MRBASE” and the R package “TwoSampleMR” (MRC Integrative Epidemiology Unit, University of Bristol).^[20]^ To evaluate the causal effect of NO_2_ air pollution exposure levels on schizophrenia risk, we used 3 models, including the IVW, IVW fixed effects, and WME models. The IVW model, which neglects the intercept in the analysis of regression, uses the inverse of outcome variance as a weight for the fit. In the IVW model, the inverse variance (i.e., Wald ratios) was determined by dividing SNP-schizophrenia associations by SNP-NO_2_ air pollution exposure level associations. The average effect of NO_2_ air pollution exposure levels on schizophrenia risk was estimated by a random effects meta-analysis of the Wald ratios. The IVW model provides accurate estimates when the inverse variance satisfies the main assumptions of MR analysis, that is, the inverse variance: is related to the exposure, does not influence the result by any routes other than the exposure, and has no relationship to the confounders.^[21]^ The WME model calculates the intercept of the fitted curve to evaluate the mean pleiotropy effect among the genetic variants. When more than half of the inverse variances meet the leading assumptions of MR analysis, the WME model can provide a conformance assessment.^[22]^ In the WME model, the causal effect is considered positive when the beta value is greater than zero and negative when the beta value is less than zero. Moreover, the causal effect is significant when the threshold *P* < .05.

### 2.5. Sensitivity analysis

To ensure the accuracy of the statistical results, we performed the horizontal pleiotropy test for instrumental variables, sensitivity analysis, and heterogeneity evaluation. The horizontal pleiotropy test was assessed with the intercept of the MR-Egger regression. If SNPs influence the schizophrenia risk through a pathway other than NO_2_ air pollution exposure, significant horizontal pleiotropy can skew the MR tests, which leads to inaccurate causal estimates.^[23]^ Sensitivity analysis was performed by removing each SNP from the original MR analysis. In the leave-one-out sensitivity analysis, we used a forest plot to express the results, which can detect whether the results were disproportionately affected by any single SNP.^[24]^ Finally, heterogeneity evaluation was performed by the IVW model, and the significant threshold was set as *P* < .05.^[25]^

## 3. Results

### 3.1. Instrumental variables selection

**Figure 2.**
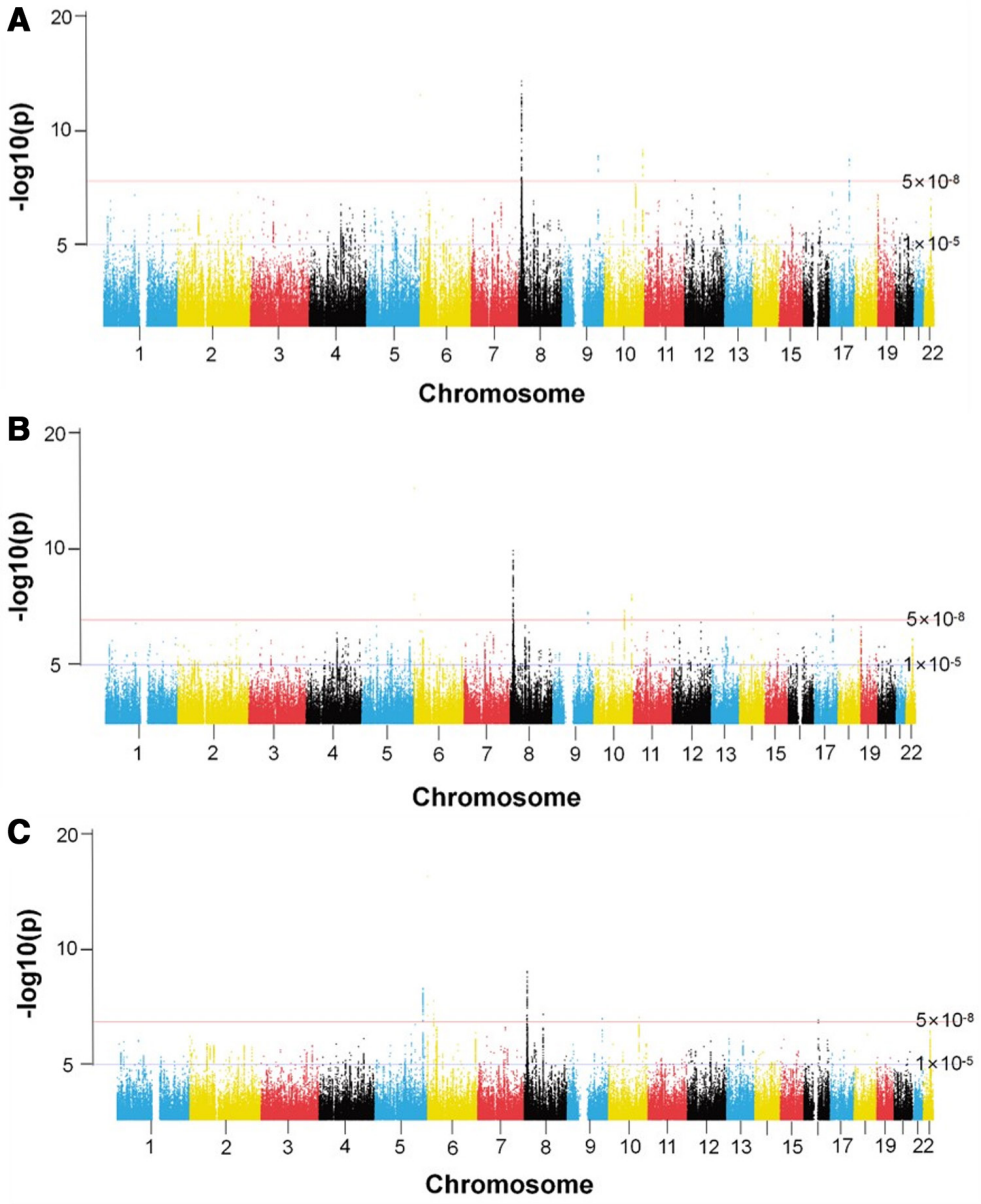
Manhattan plot of instrumental variables filtering results. The upper and lower horizontal lines in the plots indicate significance thresholds at *P* = 5 × 10^−^8 and suggestive significance at *P* = 1 × 10^−^5. Manhattan plot of instrumental variables filtering results from (A) ukb-b-2618 (139 SNPs), (B) ukb-b-5620 (128 SNPs), (C) ukb-b-9942 (102 SNPs). The corresponding instrumental variables information is listed in Table S1. SNPs = single nucleotide polymorphisms.

The NO_2_ exposure levels (exposure) and schizophrenia (outcome) used in this study were summarized data collected by the MRC Integrative Epidemiology Unit at the University of Bristol and the Psychiatric Genomics Consortium, respectively. All samples were from individuals of European ancestry. We retrieved information on SNPs (including standard error, beta, and *P* values). With the threshold value (*P* < 5 × 10^−^8) for correlation setting and LD (*R*^2^ < 0.001) for independence setting, we screened 3 sets of instrumental variables (139, 128, and 102 SNPs) through a series of quality control measures. These instrumental variables were not only significantly correlated with the NO_2_ exposure level but also independent of each other for the consortium’s schizophrenia GWAS dataset. (Fig. 2 and Table S1).

### 3.2. The causality of NO_2_ exposure level and schizophrenia disease risk

**Figure 3.**
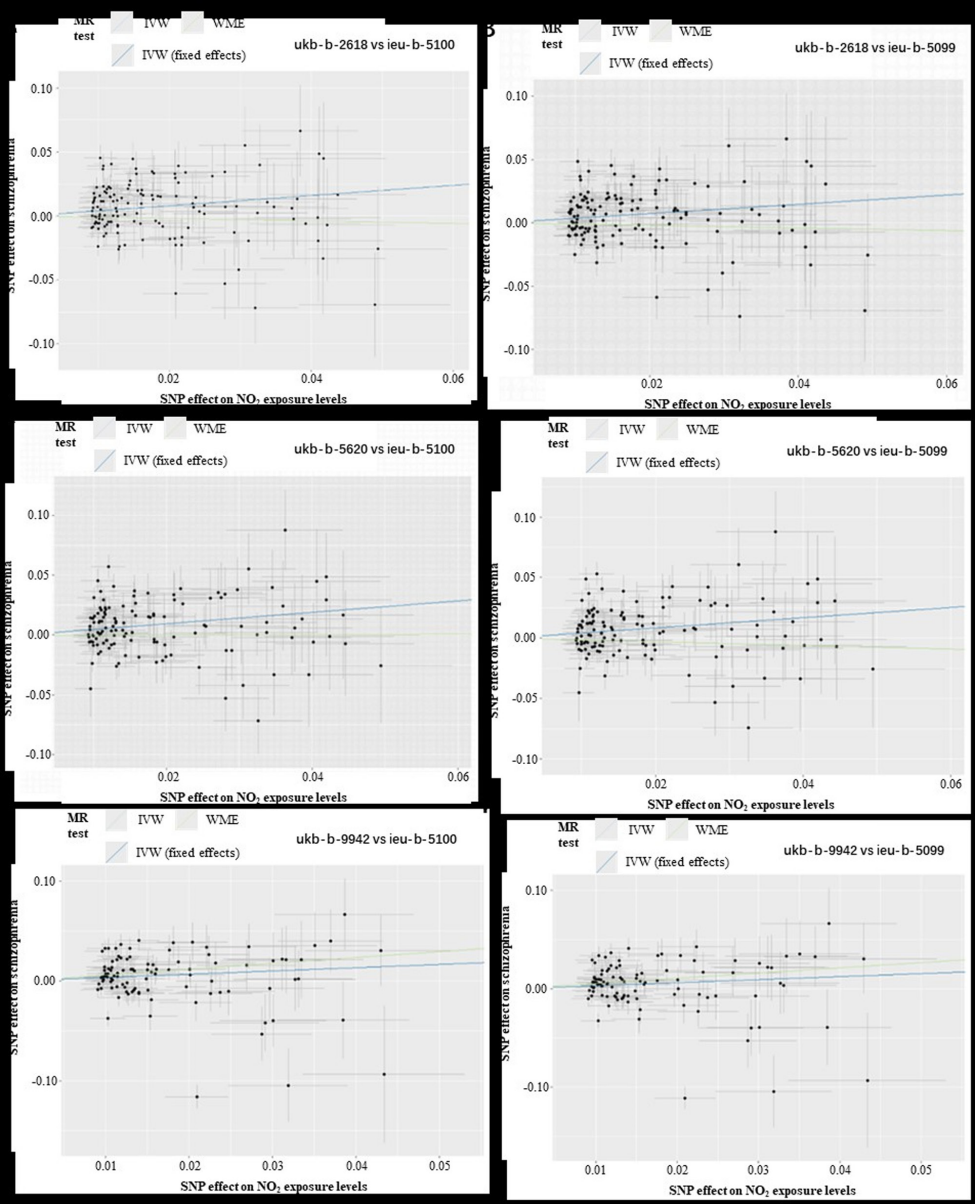
The MR analysis for the causality of NO_2_ exposure level and schizophrenia risk. The method comparison plot displays the SNP effects on schizophrenia risk versus SNP effects on NO_2_ exposure levels across the IVW, IVW fixed effects, and WME models. The slope of each line indicates the causal effect, with each line representing a different model. (A) exposure group: (ukb-b-2618) and outcome group: (ieu-b-5100); (B) exposure group: (ukb-b-2618) and outcome group: (ieu-b-5099); (C) exposure group: (ukb-b-5620) and outcome group: (ieu-b-5100); (D) exposure group: (ukb-b-5620) and outcome group: (ieu-b-5099); (E) exposure group: (ukb-b-9942) and outcome group: (ieu-b-5100); (F) exposure group: (ukb-b-9942) and outcome group: (ieu-b-5099). IVW = inverse variance weighting, MR = Mendelian randomization, NO_2_ = nitrogen dioxide, SNPs = single nucleotide polymorphisms, WME = weighted median estimator.

**Figure 4.**
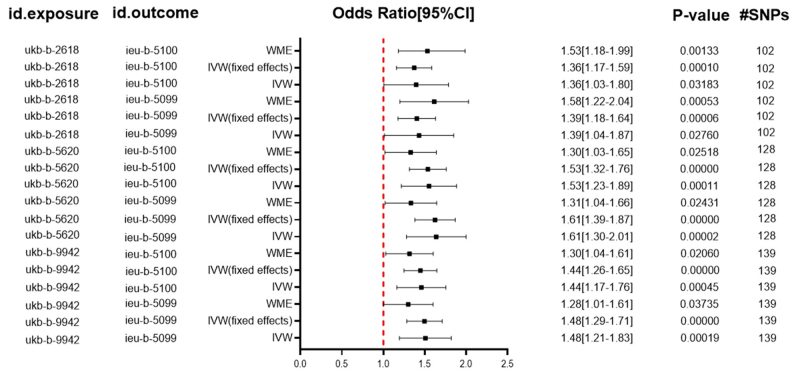
Causal associations between NO_2_ exposure levels and schizophrenia using MR analysis. The estimations were made by the IVW, IVW fixed effects, and WME models. The forest plot shows the 95% CIs of 3 types of exposure and 2 sets of output, which are indicated by the error bars. The results displayed on the forest plot are all statistically significant. CI = confidence interval, IVW = inverse variance weighting, MR = Mendelian randomization, NO_2_ = nitrogen dioxide, OR = odds ratio, WME = weighted median estimator.

Employing the 3 sets of instrumental variables (139, 128, and 102 SNPs), we conducted the 2-sample univariate MR analysis to evaluate the causal effect of NO_2_ exposure level on schizophrenia risk. Three analysis models of MR were used in this study, including 3 experimental exposure groups and 2 experimental outcome groups. Finally, we obtained 18 group analysis results, of which each model occupies 6 groups. The results of all 6 groups of the IVW model showed a significant positive causal relationship between NO_2_ exposure level and schizophrenia risk (beta = 0.3967, *P* = .0002; beta = 0.3644, *P* = .0005; beta = 0.4778, *P* = .00002; beta = 0.4236, *P* = .0001; beta = 0.3310, *P* = .0276; beta = 0.3086, *P* = .0318). The IVW fixed effects model and WME model showed similar positive results (all beta values were greater than zero, and all *P* values were < .05) (Table 1). The influence of the 3 sets of instrumental variables (139, 128, and 102 SNPs) on NO_2_ exposure level and schizophrenia in the 3 models showed good consistency (Fig. 3). Moreover, odds ratios (ORs) were calculated to quantify the effect size of genetically predicted NO_2_ exposure on schizophrenia risk. The ORs ranged from 1.30 (95% confidence interval [CI]: 1.03–1.65) to 1.60 (95% CI: 1.39–1.87), corresponding to the WME model (exposure: ukb-b-5620; outcome: ieu-b-5100) and the IVW fixed effects model (exposure: ukb-b-5620; outcome: ieu-b-5099), respectively (Fig. 4). All CIs excluded the null value (OR = 1), indicating statistically significant positive associations.

**Table 1:** The causality of NO_2_ exposure levels and schizophrenia risk by 2-sample MR.

id.exposure	id.outcome	SNPs	Model	Beta	*P* value	Horizontal pleiotropy
ukb-b-2618	ieu-b-5100	139	IVW	3.97E−01	1.94E−04	9.50E−02
ukb-b-2618	ieu-b-5100	139	IVW (fixed effects)	3.97E−01	5.00E−08	9.50E−02
ukb-b-2618	ieu-b-5100	139	WME	2.46E−01	3.74E−02	9.50E−02
ukb-b-2618	ieu-b-5099	139	IVW	3.64E−01	4.55E−04	1.80E−01
ukb-b-2618	ieu-b-5099	139	IVW (fixed effects)	3.64E−01	1.80E−07	1.80E−01
ukb-b-2618	ieu-b-5099	139	WME	2.59E−01	2.06E−02	1.80E−01
ukb-b-5620	ieu-b-5100	128	IVW	4.78E−01	2.26E−05	2.15E−01
ukb-b-5620	ieu-b-5100	128	IVW (fixed effects)	4.78E−01	1.00E−09	2.15E−01
ukb-b-5620	ieu-b-5100	128	WME	2.70E−01	2.43E−02	2.15E−01
ukb-b-5620	ieu-b-5099	128	IVW	4.24E−01	1.13E−04	3.58E−01
ukb-b-5620	ieu-b-5099	128	IVW (fixed effects)	4.24E−01	1.00E−08	3.58E−01
ukb-b-5620	ieu-b-5099	128	WME	2.67E−01	2.52E−02	3.58E−01
ukb-b-9942	ieu-b-5100	102	IVW	3.31E−01	2.76E−02	5.98E−02
ukb-b-9942	ieu-b-5100	102	IVW (fixed effects)	3.31E−01	5.92E−05	5.98E−02
ukb-b-9942	ieu-b-5100	102	WME	4.57E−01	5.33E−04	5.98E−02
ukb-b-9942	ieu-b-5099	102	IVW	3.09E−01	3.18E−02	5.04E−02
ukb-b-9942	ieu-b-5099	102	IVW (fixed effects)	3.09E−01	1.03E−04	5.04E−02
ukb-b-9942	ieu-b-5099	102	WME	4.26E−01	1.33E−03	5.04E−02

IVW = inverse variance weighting, MR = Mendelian randomization, NO2 = nitrogen dioxide, SNP = single nucleotide polymorphism, WME = weighted median estimator.

These analysis results showed that the NO_2_ exposure level increases the risk of schizophrenia.

### 3.3. Reliability check

**Figure 5.**
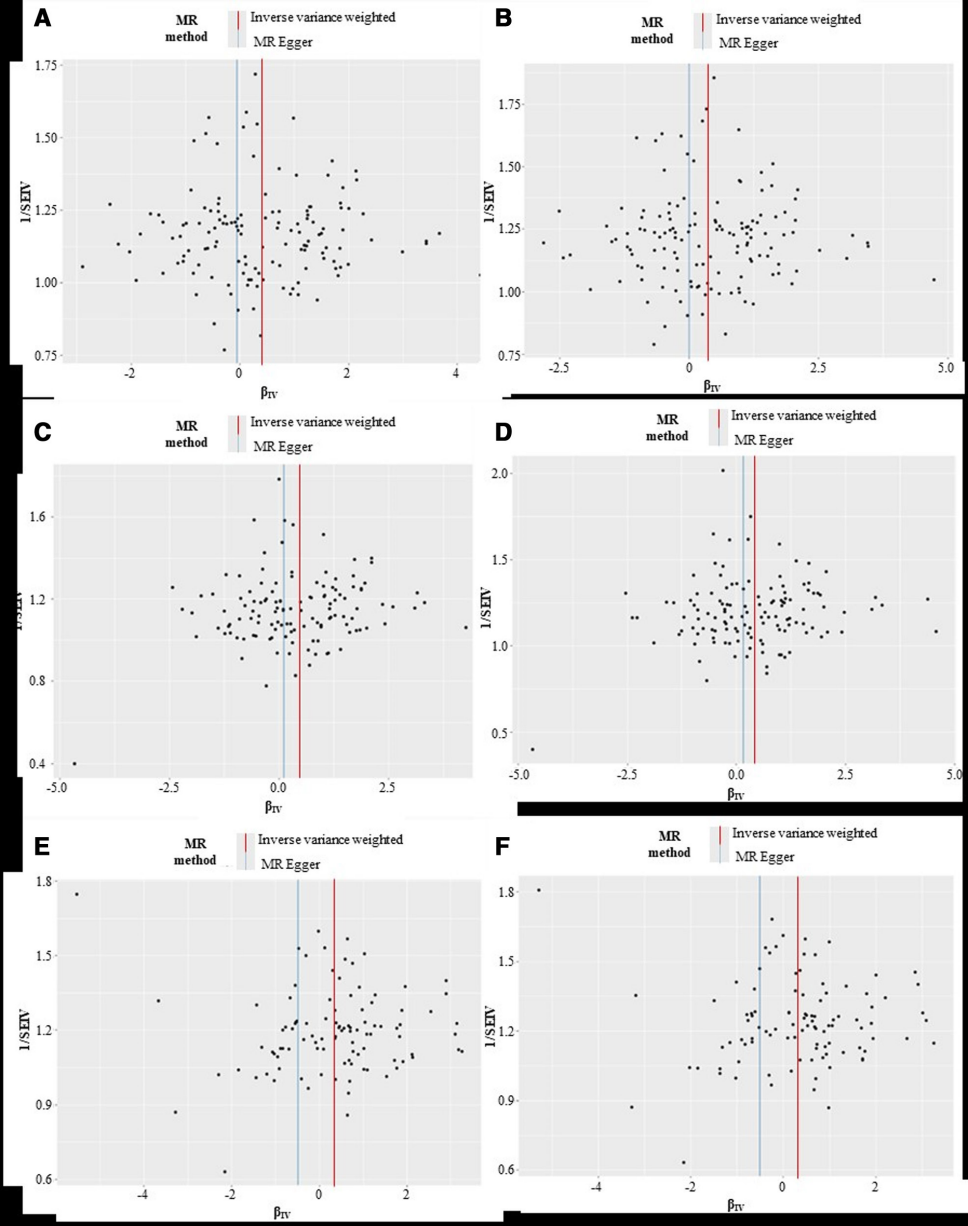
The heterogeneity evaluation of MR analysis. The funnel plot for assessing heterogeneity indicates no heterogeneity in the results of the MR analysis, which suggests that the research results are reliable. (A) exposure group: (ukb-b-2618) and outcome group: (ieu-b-5100); (B) exposure group: (ukb-b-2618) and outcome group: (ieu-b-5099); (C) exposure group: (ukb-b-5620) and outcome group: (ieu-b-5100); (D) exposure group: (ukb-b-5620) and outcome group: (ieu-b-5099); (E) exposure group: (ukb-b-9942) and outcome group: (ieu-b-5100); (F) exposure group: (ukb-b-9942) and outcome group: (ieu-b-5099). MR = Mendelian randomization.

**Figure 6.**
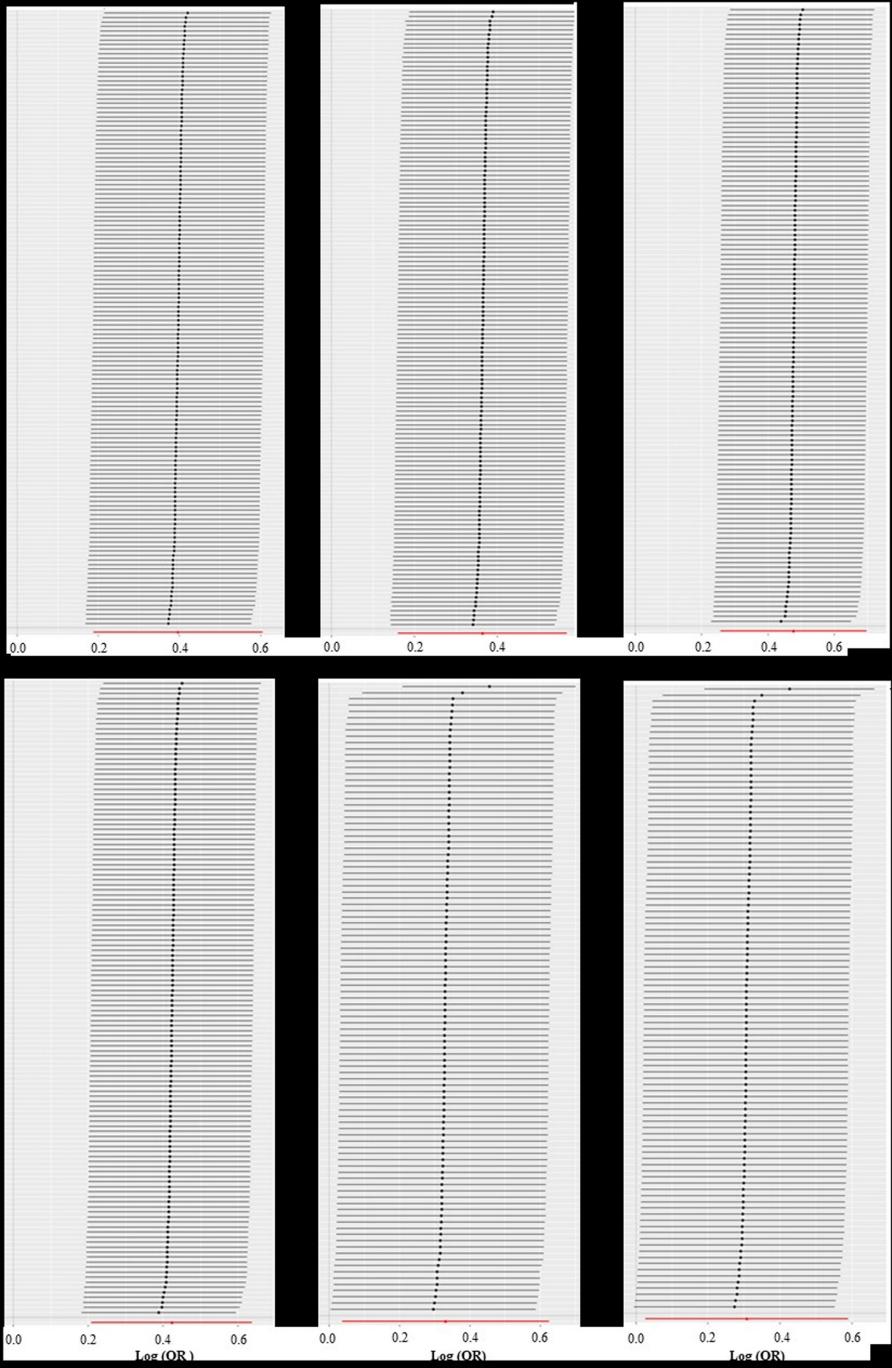
The sensitivity analysis of MR analysis. The forest plot of leave-one-out sensitivity analysis shows no heterogeneity in the MR analysis. (A) exposure group: (ukb-b-2618) and outcome group: (ieu-b-5100); (B) exposure group: (ukb-b-2618) and outcome group: (ieu-b-5099); (C) exposure group: (ukb-b-5620) and outcome group: (ieu-b-5100); (D) exposure group: (ukb-b-5620) and outcome group: (ieu-b-5099); (E) exposure group: (ukb-b-9942) and outcome group: (ieu-b-5100); (F) exposure group: (ukb-b-9942) and outcome group: (ieu-b-5099). MR = Mendelian randomization.

To ensure the accuracy of the MR statistical results, we conducted the horizontal pleiotropy evaluation for instrumental variables, heterogeneity evaluation, and sensitivity analysis. For the Psychiatric Genomics Consortium’s schizophrenia GWAS dataset, the results indicated no directional horizontal pleiotropy affecting the IVW, IVW fixed effects, and WME estimates (*P* > .05) (Table 1). Besides, Cochran Q test also indicated no heterogeneity in overall for the IVW, IVW fixed effects and WME evaluation (Fig. 5). Finally, the leave-one-out sensitivity analysis indicated that no single SNP significantly affected the MR analysis results (Fig. 6). These results confirmed that the causality of NO_2_ exposure level and schizophrenia is dependable, which further supports that NO_2_ exposure increases the risk of schizophrenia.

## 4. Discussion

Our study demonstrates that genetically predicted NO_2_ exposure has a positive causal effect on schizophrenia risk. Across 3 exposure groups and 2 outcome datasets, MR analyses using IVW, IVW fixed effects, and WME models consistently indicated positive causal effects, with ORs ranging from 1.30 to 1.60, and all CIs above unity, suggesting a modest but robust increase in risk.

Schizophrenia is a complex psychiatric disorder influenced not only by genetic factors but also by environmental exposures, including air pollutants such as NO_2_, which often coexist with PM_2.5_ and PM_10_.^[11,26,27]^ Existing evidence indicates that NO_2_ coexists with PM_2.5_ and PM_10_ in the air and can adversely affect the central nervous system through multiple pathways. For example, previous studies in mice demonstrated that NO_2_ exposure impairs cognitive function and induces neurotoxic changes, potentially via cyclooxygenase-mediated arachidonic acid metabolism.^[28,29]^ On the other hand, modulation of the endocannabinoid system, such as increasing 2-arachidonoylglycerol levels by inhibiting monoacylglycerol lipase, can mitigate NO_2_-induced neuroinflammation and neuronal damage, highlighting the regulatory role of lipid signaling in maintaining neuronal homeostasis.^[28]^ Other studies indicated that NO_2_ can disrupt insulin signaling pathways in the central nervous system, leading to activation of the mitogen-activated protein kinase signaling pathway in the brain. This disruption impairs downstream insulin receptor substrate-1/protein kinase B/glycogen synthase kinase-3β signaling, alters tau protein phosphorylation, and reduces synaptic protein expression, ultimately affecting the development of tau pathology.^[30]^ Collectively, these mechanisms provide a biologically plausible link between NO_2_ exposure and schizophrenia risk.

Previous epidemiological studies have reported that long-term exposure to NO_2_-containing air pollution increases schizophrenia risk by approximately 10 to 20%, based on longitudinal studies in European and North American populations.^[12,14,16,31]^ However, the evidence remains variable, highlighting the need for causal inference analyses. Our MR study not only reinforces these observations but also provides additional genetic evidence supporting a causal relationship between NO_2_ exposure and schizophrenia.

Although this study has limitations, including restriction to European ancestry and the use of summary-level GWAS data, the overall findings appear robust. Despite these constraints, the consistent results across multiple MR models support a causal role of NO_2_ exposure in increasing schizophrenia risk and highlight the environmental contribution to its etiology. Overall, this work enhances our understanding of the potential pathophysiological impact of NO_2_ on the central nervous system and underscores the environmental contribution to schizophrenia etiology.

## 5. Conclusion

Our study provides genetic evidence supporting a causal association between NO_2_ exposure and increased schizophrenia risk. The consistent results across multiple MR models strengthen the robustness of this finding. These results highlight the potential pathophysiological impact of NO_2_ on the central nervous system and underscore the importance of environmental factors in the etiology of schizophrenia. Further studies in diverse populations would provide valuable insight.

## Author contributions

**Conceptualization:** Xiao Zou.

**Data curation:** Chunying Li.

**Formal analysis:** Chunying Li.

**Funding acquisition:** Xiao Zou.

**Methodology:** Chunying Li.

**Resources:** Chunying Li.

**Software:** Chunying Li.

**Supervision:** Xiao Zou.

**Validation:** Chunying Li.

**Visualization:** Chunying Li.

**Writing – original draft:** Chunying Li.

**Writing – review & editing:** Xiao Zou.


